# Between thrombosis and bleeding: the clinical dilemma of anticoagulation after intracranial hemorrhage

**DOI:** 10.1097/MS9.0000000000004923

**Published:** 2026-04-03

**Authors:** Aime Ishimwe Mugisha

**Affiliations:** School of Medicine and Pharmacy, College of Medicine and Health Sciences, University of Rwanda, Kigali, Rwanda

*To the Editor*,

Intracranial hemorrhage (ICH) accounts for approximately 10–15% of all strokes, yet contributes disproportionately to stroke-related mortality and long-term disability^[^1^]^. Survivors of acute ICH frequently require prolonged immobilization and intensive care, conditions that substantially increase the risk of venous thromboembolism (VTE). Reported VTE incidence after ICH ranges from 5% to 15%, with pulmonary embolism remaining a preventable cause of in-hospital death^[^2^]^. The decision to initiate anticoagulation in this context represents a recurring neurocritical dilemma: preventing potentially fatal thrombosis while avoiding exacerbation of intracranial bleeding.

Several cohort studies have demonstrated that patients with spontaneous ICH face a higher risk of VTE compared with ischemic stroke, particularly within the first 2 weeks following hemorrhage^[^3^]^. Contributing mechanisms include immobility, endothelial injury, inflammatory activation, and coagulation cascade dysregulation. Mechanical prophylaxis alone, although widely recommended in the acute phase, has shown limited efficacy in preventing deep vein thrombosis when used as monotherapy^[^4^]^. Consequently, pharmacologic anticoagulation remains an essential yet controversial intervention.

The principal concern limiting anticoagulant use after ICH is hematoma expansion, a strong predictor of poor neurological outcome. Early hematoma growth occurs in up to one-third of patients, most commonly within the first 24–48 h^[^5^]^. Anticoagulation during this vulnerable period is traditionally avoided due to fear of worsening intracranial bleeding. However, accumulating evidence suggests that low-dose anticoagulation initiated after radiographic stability may not significantly increase the risk of hemorrhage progression. A large meta-analysis reported no significant difference in hematoma expansion or mortality among patients receiving prophylactic-dose heparin after documented hematoma stability compared with those receiving mechanical prophylaxis alone^[^6^]^.

Timing remains the most contentious aspect of anticoagulation after ICH. Current guidelines offer cautious recommendations, often suggesting initiation of low-dose anticoagulation between 24 and 72 h after hemorrhage onset once repeat imaging confirms stability^[^1,7^]^. Nevertheless, these recommendations are largely derived from observational data and expert consensus rather than randomized controlled trials. Variability in hemorrhage location, volume, etiology, and patient comorbidities further complicates standardized decision-making.

Patient selection is equally critical. Individuals with large hematomas, intraventricular extension, uncontrolled hypertension, or underlying coagulopathies may carry a substantially higher bleeding risk. Conversely, patients with prior VTE, active malignancy, prolonged immobility, or severe systemic inflammation may derive greater benefit from earlier anticoagulation^[^8^]^. The absence of validated risk stratification tools forces clinicians to rely on individualized judgment, leading to heterogeneous practices across institutions.

The clinical consequences of untreated VTE can be profound. Pulmonary embolism contributes significantly to early mortality after ICH and may negate otherwise favorable neurological recovery^[^2^]^. Conversely, hemorrhagic progression may result in irreversible brain injury or death. This balance between competing risks reflects a broader challenge in neurocritical care, where interventions aimed at preventing systemic complications may directly influence neurological outcomes.

In light of these uncertainties, future research must prioritize prospective, adequately powered trials to define optimal anticoagulation timing, dosing, and patient selection after ICH. Incorporation of advanced imaging markers, biomarkers of coagulation and inflammation, and individualized risk models may enable safer, more precise decision-making. Until such data are available, anticoagulation after ICH should remain a carefully individualized decision, balancing thrombotic risk against hemorrhagic vulnerability rather than adhering to uniform protocols. The letter to the editor follows the guidance of the Transparency in the Reporting of Artificial Intelligence in Research (TITAN) guideline^[^9^]^.

## Data Availability

Not applicable.
